# The consequences of social category faultlines in high- and low-context cultures: A comparative study of Brazil and Germany

**DOI:** 10.3389/fpsyg.2022.1082870

**Published:** 2022-12-22

**Authors:** Kathrin Burmann, Thorsten Semrau

**Affiliations:** Professorship of Management, Trier University, Trier, Germany

**Keywords:** team composition, faultlines, gender, age, task conflict, high- and low-context cultures, team performance

## Abstract

The present study sheds light on how differences between high- and low-context cultures influence the consequences of social category faultlines. To develop our theoretical arguments, we integrate ideas from faultline theory and Hall’s theory on cultural contexts. We test our hypotheses using survey data from 54 teams in the banking industry in Germany, a nation with a low-context culture, and in Brazil, a country with a high-context culture. In line with our theorizing, the study results reveal that whether social category faultline strength stimulates task conflict and is thus detrimental to team performance depends on the societal culture in which teams operate. Specifically, we observe that social category faultlines stimulate task conflict and thus have a negative indirect effect on team performance in Germany’s low-context culture, while we find no such effects in the high-context culture of Brazil. We discuss the theoretical and practical implications of our study and close with some suggestions for future research.

## 1. Introduction

Due to the increasing diversity of the workforce (Van Knippenberg and Schippers, 2007; Wegge et al., 2008) and the still-growing popularity of teams as basic work units within organizations (DeShon et al., 2004; Stewart, 2006), the question of how demographic diversity may influence team processes and outcomes is of considerable interest for researchers and practitioners alike.

When elaborating on the consequences of demographic diversity, researchers have traditionally focused on team heterogeneity with respect to *single* demographic attributes, such as age *or* gender. Unfortunately, the cumulative findings resulting from this research are largely inconclusive (Horwitz and Horwitz, 2007; Bell et al., 2011; Van Dijk et al., 2012). Thus, scholars have begun to address the more complex compositional patterns of team demographic diversity and have investigated the distribution of *multiple* demographic attributes simultaneously (Thatcher and Patel, 2011; Thatcher and Patel, 2012). Specifically, over the past decades, there has been an increasing interest in team demographic faultlines, i.e., the alignment of demographic attributes that may split a team into dissimilar, homogenous subgroups (Lau and Murnighan, 1998; Thatcher and Patel, 2012). Building on this conceptualization and drawing on ideas from social categorization theory (Tajfel, 1981; Turner, 2010) and the similarity attraction paradigm (Byrne, 1971), prior research has established that demographic faultlines are inherently conflictual (Bezrukova et al., 2012) and can thus be detrimental for team performance (Thatcher and Patel, 2011; Thatcher and Patel, 2012).

However, our understanding of the consequences of demographic faultlines is still limited (Pregernig, 2017; Antino et al., 2019). Specifically, prior faultline research has largely overlooked the importance of the environmental contexts in which teams operate (Bezrukova et al., 2012; Cooper et al., 2014; Wu et al., 2021). Given that teams are open systems and thus subject to influences emanating from their environment (Mathieu et al., 2008a), this is a significant shortcoming. We contribute to closing this gap in the literature by investigating how differences in the societal cultural context in which teams are embedded shape the consequences of social category faultlines, i.e., faultlines based on the distribution of age *and* gender within teams (Bezrukova et al., 2009). Paying attention to a potential interplay between social category faultlines and societal culture seems fruitful for several reasons. First, cultural norms and values influence behavior in organizations and team interaction patterns by showing appropriate ways of relating to others (House et al., 2004). Second, prior research on gender heterogeneity suggests that societal culture can affect the consequences of demographic differences within teams, as it shapes social categorization processes and influences how individuals deal with members from different demographic subgroups (Schneid et al., 2015).

To develop our theoretical arguments, we integrate ideas from faultline theory (Lau and Murnighan, 1998), theory on high- and low-context cultures (Hall, 1976), and the input-process-outcome model of team performance (McGrath, 1984). Specifically, we build on faultline theory (Li and Hambrick, 2005; Bezrukova et al., 2007; Thatcher and Patel, 2011) to first suggest that the intellectual differences resulting from social category faultlines stimulate task conflict. Second, drawing on how high- and low-context cultures shape behavior and social interactions (Hall, 1976; Kim et al., 1998), we argue that the consequences of social category faultline strength for team task conflict will likely differ across high- and low-context cultures. Third, based on the arguments established before and the widely accepted input-process-outcome framework of team performance (McGrath, 1984), we submit that there will be an indirect relationship between social category faultline strength and team performance *via* task conflict, which is moderated by societal culture.

We test our ideas based on data from 282 employees working in 54 teams in the banking industry in Germany—a low-context culture (Hall, 1976; Rosenbloom and Larsen, 2003)—and in Brazil—a high-context culture (Rosenbloom and Larsen, 2003; Sobral et al., 2008). The results provide support for our theoretical arguments.

With the insights generated, the present study contributes by extending our knowledge in several ways. We answer scholarly calls to shed light on the interplay between faultlines and culture (Gibson and Vermeulen, 2003; Bezrukova et al., 2012; Wu et al., 2021). In identifying societal culture in which teams are embedded as an important boundary condition for the consequences of social category faultlines, we complement prior research elaborating on the relevance of other contextual characteristics, such as the cultural alignment between the team and the department in which it is embedded (Bezrukova et al., 2012) as well as an organization’s industry environment (Cooper et al., 2014; Wu et al., 2021). By revealing that whether social category faultlines stimulate task conflict within teams is contingent upon the cultural context in which teams operate, our study further contributes to an ongoing debate on whether social category faultlines are a driver of task conflict within teams (Choi and Sy, 2010; Thatcher and Patel, 2011).

Our study also informs the literature on task conflict (Jehn, 1995) and on high- and low-context cultures (Mintu-Wimsatt and Gassenheimer, 2000; Mintu-Wimsatt, 2002). By showing that faultlines can serve as a driver of task conflict, our study contributes to a better understanding of the role of team composition in task conflict emergence (Van Knippenberg and Schippers, 2007). Indicating that the task conflict emanating from social category faultlines is detrimental for team performance, our findings also contribute to the discussion on the performance implications of task conflict in teams (De Dreu and Weingart, 2003; De Wit et al., 2012). In highlighting that the indirect performance effect of social category faultlines *via* task conflict is affected by differences in societal culture, our study further adds to our understanding of whether the link between task conflict and important outcomes is specific to culture (Nibler and Harris, 2003; Bisseling and Sobral, 2011). Our study complements prior research on Hall's (1976) context theory by showing that differences between high- and low-context cultures affect the consequences of team composition. Given that social category diversity is a team input characteristic that can be manipulated through selection and placement (Bell, 2007), we believe that our study also has important practical implications.

## 2. Literature review and hypotheses

Traditionally, research on team demographic diversity has focused on the effects of heterogeneity with respect to single demographic attributes, such as age *or* gender, on important work outcomes (Bell et al., 2011). However, both positive and negative consequences of demographic diversity for team processes and outcomes have been theorized and observed empirically, leading to cumulative findings that are largely inconclusive (Thatcher and Patel, 2011).

Building on and expanding prior conceptualizations of demographic diversity (Blau, 1977; O'Reilly III et al., 1989), Lau and Murnighan (1998) introduced the concept of group faultlines, which describes how the compositional dynamics of multiple demographic attributes, such as age *and* gender, can potentially subdivide a team. The following example illustrates the difference between the traditional view on diversity and the faultline perspective: There are two teams with four members each. Team 1 comprises two men aged 50 and two women aged 20. Team 2 comprises one man and one woman aged 50 and one man and one woman aged 20. From the traditional diversity perspective, both teams are identical. With two men and two women, both teams have the same level of gender diversity, and with two individuals who are 50 years of age and two individuals who are 20 years of age, both teams also have the same level of age diversity. From a faultline perspective, however, the two teams differ significantly because patterns of age and gender diversity align in Team 1 but not in Team 2. In Team 1, gender-related differences between team members converge with age-related differences because both team members who are 50 years of age are male and both team members who are 20 years of age are female. In contrast, there is no such convergence in Team 2. While the two teams are thus virtually identical based on the traditional view on diversity, the faultline perspective suggests that based on processes of social categorization related to visible and accessible attributes (Tajfel, 1981; Turner, 2010), the members of Team 1 will more likely categorize themselves into different subgroups than the members of Team 2.

Based on the seminal work of Lau and Murnighan (1998) and drawing on social identity and self-categorization theories (Tajfel, 1981; Turner et al., 1987), scholars have elaborated on the consequences of demographic faultline strength, i.e., the extent to which the alignment of demographic attributes within a team fuels the formation of homogeneous subgroups (Thatcher and Patel, 2011). Overall, and in line with predictions made by the categorization-elaboration model (Van Knippenberg et al., 2004), research has found that strong faultlines are typically dysfunctional (Bezrukova et al., 2012; Thatcher and Patel, 2012), as they impede effective group functioning (Molleman, 2005), creativity (Pearsall et al., 2008), and team satisfaction and performance (Thatcher and Patel, 2011). Research has also elaborated on how these consequences might be explained. Specifically, and in line with the idea that while faultlines result in team members having pleasant interactions with members of their own subgroup, they also result in competition (Halevy, 2008) and communication hindrances (Lau and Murnighan, 2005) across subgroups, meta-analytical evidence suggests that faultlines often result in task conflict within teams (Thatcher and Patel, 2011), which is a threat to teamwork (Bezrukova et al., 2007; Thatcher and Patel, 2011) and may escalate to relationship conflict (Simons and Peterson, 2000; Yang and Mossholder, 2004; Mooney et al., 2007; Gamero et al., 2008).

While faultline scholars have thus far predominantly elaborated on the direct and indirect effects of faultline strength, a stream of research has begun to explore contingencies that can qualify these effects (Thatcher and Patel, 2012). Specifically, previous studies have identified team member characteristics (Homan et al., 2008), task-related variables such as task autonomy (Rico et al., 2007), and team leader behavior (Kunze and Bruch, 2010) as contingencies for the effects of demographic faultline strength. In contrast, the potential impact of a team’s external environmental context, i.e., the organizational, industrial, and societal conditions in which teams operate (Schneid et al., 2015), has thus far been largely overlooked (Bezrukova et al., 2012; Cooper et al., 2014). The few notable exceptions include a study conducted by Bezrukova et al. (2012), which provides evidence suggesting that cultural alignment between a team and the department in which it is embedded can affect the consequences of faultlines for team performance. Similarly, studies conducted by Cooper et al. (2014) and Wu et al. (2021) reveal that the effect of faultlines can vary with the characteristics of the industry environment in which teams and their organizations operate.

**Figure 1:**
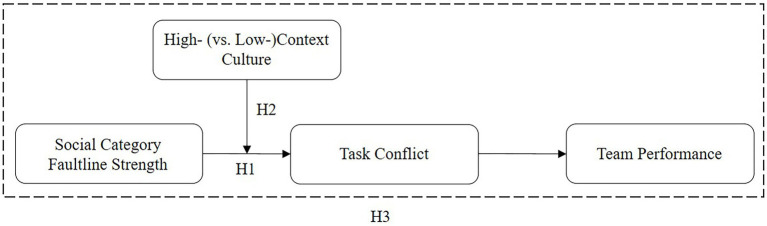
Conceptual model.

To expand our knowledge on the relevance of environmental context characteristics for faultline consequences, the present study complements this prior research by paying special attention to the societal culture in which teams are embedded. We first develop detailed arguments to suggest why social category faultline strength stimulates team task conflict (H1). Then, we explain why we expect this effect to be moderated by the societal culture in which teams are embedded, i.e., whether they operate in a high-context or low-context culture (H2). Building on the arguments established, we finally hypothesize that contingent upon societal culture, there will be an indirect negative relationship between social category faultline strength and team performance *via* by task conflict (H3). Figure 1 shows our conceptual model.

### 2.1. Social category faultline strength and task conflict

It has been widely acknowledged that faultlines are inherently conflictual (Bezrukova et al., 2012). In line with this notion and building on prior research (Li and Hambrick, 2005; Bezrukova et al., 2007; Thatcher and Patel, 2011), we delineate why we expect social category faultline strength to engender task conflict, i.e., disagreements about task-related ideas, methods, and judgments within a team (Jehn, 1995; De Dreu and Weingart, 2003; De Wit et al., 2012).

As described above, faultline strength increases the probability of subgroups with clear within-group similarities and between-group differences emerging within teams (Lau and Murnighan, 1998). As such, faultline strength facilitates intellectual opposition within a team (Li and Hambrick, 2005). When dissimilar subgroups emerge, members of different subgroups harbor divergent frames of reference and develop different ideas on how to approach and solve task-related problems (Brewer, 1991; Thatcher and Patel, 2011). As a result, the probability of task-related disagreements within teams increases with faultline strength. Faultline strength further fuels task conflict by facilitating polarization and competitive clashes between subgroups and increasing the probability of even the most controversial ideas being expressed, advocated, and vehemently defended (Bezrukova et al., 2007; Nishii and Goncalo, 2008; Thatcher and Patel, 2011). Due to mutual sympathy and perceived similarities among aligned members, faultlines foster solidarity and mutual support among subgroups (Stevenson et al., 1985; Lau and Murnighan, 2005; Bezrukova et al., 2007). This provides subgroup members with the confidence needed to openly express and defend ideas and perspectives, even when these are clearly at odds with what members of other subgroups believe and desire (Bezrukova et al., 2007; Nishii and Goncalo, 2008). While faultline strength increases tendencies among team members to conform to the ideas and opinions favored by their own subgroup (Baron et al., 1992; Bezrukova et al., 2007; Jehn et al., 2008), it also results in team members distancing themselves from the views and perspectives of other subgroups (Brewer et al., 1993; Van Knippenberg et al., 2004). As a result, subgroups polarize around their ideas and thoughts, which they will strongly advocate and defend, while vehemently opposing ideas suggested by nonsubgroup members (Brewer, 1991; Bartel, 2001), resulting in competitive clashes that cannot be easily resolved (Clark et al., 2000).

Based on these arguments and in line with prior research (Li and Hambrick, 2005; Bezrukova et al., 2007; Thatcher and Patel, 2011), we expect faultline strength to stimulate task conflict within teams. Thus, we propose the following:

*Hypothesis 1 (H1)*: There is a positive relationship between social category faultline strength and task conflict.

### 2.2. Societal culture as a moderator

While the above lines of reasoning suggest a positive relationship between social category faultline strength and task conflict, we predict the magnitude of this link to be contingent upon the societal culture in which teams are embedded.

Societal cultures vary considerably with respect to prevalent cultural norms and values, which shape individual perceptions and behavior in organizations and can influence team interaction patterns (Hall, 1959; O'Reilly, 1989; Schwartz, 1999; House et al., 2004). In line with this notion, prior research suggests that in shaping social categorization processes and the inclusion of individuals from different social categories, differences in societal culture can influence the consequences of demographic differences within teams (Schneid et al., 2015). Building on this idea, in this section, we develop arguments to suggest why the effects of social category faultline strength on task conflict likely differ across high- and low context cultures (Hall, 1976).

Low-context cultures emphasize individualism, directness and detachment (Hall, 1976). In such societal cultures, individuals tend to be uncompromising (Hall, 1976; Kim et al., 1998) and express their criticism directly (Würtz, 2005). When strong rather than weak social category faultlines provide them with loyalty and support from their subgroup, individuals in low-context cultures will likely feel inclined to openly express ideas and perspectives, even when these run counter to what members of other subgroups consider appropriate. Given that individuals in low-context cultures tend to be uncompromising (Hall, 1976; Kim et al., 1998), competitive clashes between subgroups resulting from intense polarization are also highly probable (Insko et al., 1990). Thus, in low-context cultures, strong faultlines will likely result in high levels of task conflict.

In high-context cultures, in contrast, intellectual differences emanating from faultline strength will less likely fully translate into task conflict. High-context cultures emphasize conformity (Hall, 1976; Kim et al., 1998) and discourage open confrontation (Kim et al., 1998). Thus, in high-context cultures, individuals tend to repress their own feelings and interests to maintain harmony and close relationships with others (Hall, 1976; Kim et al., 1998). In such a context, task conflict emanating from the divergent ideas and frames of reference associated with social category faultlines are less likely to become very intense. Given prevalent norms and values, individuals in high-context cultures tend to be more agreeable and less confrontational (Hall, 1976; Mintu-Wimsatt and Gassenheimer, 2000; Würtz, 2005) and to avoid overt conflict even when there are serious differences in opinion (Hall and Hall, 1990; Kim et al., 1998). Thus, to preserve harmony, team members in high-context cultures will be more likely to refrain from expressing controversial ideas and perspectives even when mutual liking and perceived similarity provide them with approval support of their subgroup. For similar reasons, team members embedded in a high-context culture are less likely to behave in a highly assertive and competitive manner when either defending ideas originating from their own subgroup or opposing ideas originating from nonsubgroup members, which decreases the probability of intense competitive clashes between subgroups (Insko et al., 1990).

Based on these arguments, we expect the positive link between task conflict and social category faultline strength to be moderated by the societal cultural context in which teams operate and to be weaker in a high-context culture than in a low-context culture. We thus hypothesize the following:

*Hypothesis 2 (H2)*: Societal culture moderates the positive relationship between social category faultline strength and task conflict such that this relationship will be weaker in a high-context culture than in a low-context culture.

### 2.3. Societal culture and the indirect relationship between social category faultline strength and team performance

Team performance has been heavily investigated as an important outcome that can be affected by faultline strength (Thatcher and Patel, 2011). When trying to explain the negative effect of faultline strength on performance that is typically observed, scholars have pointed to the fact that faultlines impair group functioning because they are inherently conflictual (Thatcher and Patel, 2011; Bezrukova et al., 2012). In line with these notions and building on the above arguments suggesting that while social category faultline strength increases task conflict (H1), the magnitude of this effect will vary with the societal culture in which teams are embedded (H2), we subsequently elaborate on why we expect an indirect relationship between social category faultline strength and team performance *via* task conflict, which is contingent upon the societal culture in which teams operate (H3). In developing our theoretical reasoning, we adopt the widely accepted framework of the input-process-outcome model of team performance (McGrath, 1984), which suggests that team processes—such as task conflict—serve as mediating variables (Mathieu et al., 2008b) that help explain how crucial team inputs are transformed into important outcomes (Mathieu et al., 2008a).

Originally, task conflict was thought to be a source of creativity and informed decision making, thus enhancing team performance (Jehn, 1995). However, studies on the link between task conflict and performance could not substantiate this claim. In fact, meta-analytical evidence either shows task conflict to be negatively related to team performance (De Dreu and Weingart, 2003) or indicates that task conflict may have no substantial performance effect (De Wit et al., 2012). While the overall connection between task conflict and team performance is thus still somewhat inconclusive, scholars have emphasized that for several reasons, task conflict emanating from faultlines is likely detrimental for team performance (Li and Hambrick, 2005; Bezrukova et al., 2007; Jehn and Bezrukova, 2010). Task conflict resulting from faultlines increases team members’ cognitive load (Bezrukova et al., 2007), which can interfere with creativity and complex thinking and deplete team members’ resources needed for task completions and meeting team goals (Jehn and Bezrukova, 2010). Task conflict resulting from subgroup emergence, polarization, and competitive clashes within a team further hinders team performance, as it stimulates feelings of tension and discomfort (Li and Hambrick, 2005; Bezrukova et al., 2007; Choi and Sy, 2010). Given that such feelings increase stress (Yang and Mossholder, 2004; Dijkstra et al., 2005) and can result in member dissatisfaction and withdrawal (Li and Hambrick, 2005), task conflict emanating from faultline strength further impedes team decision-making and effectiveness.

Thus, based on these arguments and building on the arguments leading to H1 and H2, we expect a negative indirect effect of social category faultline strength on team performance *via* task conflict, which is contingent on the societal culture in which teams operate. Specifically, we suggest that the negative indirect link between social category faultline strength and team performance *via* task conflict is weaker in a high-context culture than in a low-context culture. Thus, we hypothesize the following:

*Hypothesis 3 (H3)*: There is a negative indirect relationship between social category faultline strength and team performance via task conflict moderated by societal culture such that this relationship is weaker in a high-context culture than in a low-context culture.

## 3. Materials and methods

### 3.1. Setting and sample

To test our hypotheses, we collected data from teams operating in a high-context culture and from teams operating in a low-context culture. Specifically, we collected data from Brazil, which prior research (Rosenbloom and Larsen, 2003; Sobral et al., 2008) has identified as a high-context culture. Research suggests that the societal culture in Brazil values cordiality (Lourenção et al., 2019) and close interpersonal relations (Sobral et al., 2008), whereby Brazilians tend to maintain harmony and avoid open and direct confrontation (Sobral et al., 2008). Complementarily, we collected data from Germany, which is considered a low-context culture (Hall, 1976; Rosenbloom and Larsen, 2003), as German society emphasizes individualism (Hall, 1976) and Germans tend to prefer direct and open communication (Takhtarova et al., 2019).

To alleviate potential concerns related to the internal validity of our study, we cooperated with organizations from one particular industry to collect our data in the two national contexts. Specifically, we cooperated with a German bank and a Brazilian bank and invited their employees working in teams as well as their respective team leaders to participate in our study. Building on prior research (Offermann and Spiros, 2001), we focused on teams with fewer than 20 members to ensure that team members had joint responsibilities. Team members provided demographic data allowing us to calculate social category faultline strength as well as information on task conflict and on several of our controls. Team leaders reported on team performance.

In total, 402 team members (227 from Brazil and 175 from Germany) participated in our study, resulting in a response rate of 53.67%. In total, 86 team leaders (51 from Brazil and 35 from Germany) completed our survey, resulting in a response rate of 85.15%. We excluded teams with unmatched responses from team leaders and team members. To ensure the reliability and validity of our data, we followed earlier conflict research (Chun and Choi, 2014) by excluding teams with low within-team agreement on task conflict, i.e., mean *r*_wg(j)_ values of lower than .50, and with team-level response rates of less than 50%. As a result, our final sample includes data for 54 teams (22 teams from Brazil; 32 from Germany) comprising information provided by 282 employees and 54 team leaders. On average, the teams in our sample included 6.76 members (*SD* = 3.77), and team members had worked on their respective teams for 8.40 years (*SD* = 8.29). Team members were, on average, 39.45 years old (*SD* = 12.34). In total, 62.77% of the team members were female.

### 3.2. Measures

We relied on established scales to capture our study variables. To ensure contextual equivalence, all items originally available in English were translated into German and Portuguese and then back-translated by accredited translators following the procedure described by Brislin (1970). To ensure comprehensibility, we pretested our survey with respondents from the field who did not participate in the main study (Sudman et al., 1996). As described in detail below, we followed established recommendations (Schaffer and Riordan, 2003; Hult et al., 2008) to ensure measurement equivalence across national contexts by conducting multigroup CFAs (Byrne, 2009) for our main study variables.

*Team performance.* To capture team performance, we followed earlier research (Kirkman and Rosen, 1999; Barrick et al., 2007; Chen et al., 2011; Semrau et al., 2017; Kim et al., 2021) and used the 6-item scale developed by Kirkman and Rosen (1999). A sample item is “This team meets or exceeds its goals.” Team leaders indicated their agreement on a five-point Likert-type scale ranging from 1 (*strongly disagree*) to 5 (*strongly agree*). The six items showed a high level of consistency (Cronbach’s α = 0.853). To ensure the cross-national validity of our team performance measure, we utilized a two-step approach. First, we analyzed the fit of our measurement model with factor loadings that were constrained across the national context. The results indicated a very good overall fit with our data (χ^2^ = 19.563; df = 19; CFI = 0.996; RMSEA = 0.024). In a second step, we employed a χ^2^ difference and a CFI difference test (Cheung and Rensvold, 2002; Byrne, 2009) to compare our measurement model to a model with unconstrained factor loadings. Indicating equivalence, both tests [∆χ^2^_(5)_ = 8.206, n.s. and ∆CFI = 0.004] revealed that the unconstrained baseline model did not provide a better fit with our data.

*Social category faultline strength.* In line with prior research (Bezrukova et al., 2009; Choi and Sy, 2010), we examined social category faultline strength based on information on age and gender provided by the team members. To compute our measure of social category faultline strength, we relied on the average silhouette width (ASW) algorithm developed by Meyer and Glenz (2013).^1^ The ASW algorithm uses a two-stage cluster analysis approach to combine team heterogeneity values related to multiple demographic attributes into one indicator of social category faultline strength (Bahmani et al., 2018). In the first step, the algorithm employs a hierarchical cluster analysis to identify the initial set of subgroups for a given team. In the second step, the algorithm permutes and rearranges team members across subgroups to identify the subgroup constellation with the strongest faultline. The resulting ASW values, ranging from 0 to 1, indicate social category faultline strength (Bahmani et al., 2018; Tiede et al., 2021).

*Task conflict.* We assessed task conflict based on team members’ conflict perceptions captured with three items adapted from Jehn and Mannix (2001). A sample item is “How often do you have competing ideas in your team?” Team members indicated their responses using a five-point Likert-type scale ranging from 1 (*never*) to 5 (*always*). The three items showed a high level of consistency (Cronbach’s α = 0.813). Again, we utilized a two-step approach to ensure the cross-national validity of our measure. Goodness-of-fit indicators demonstrated a good overall fit of our measurement model (χ^2^ = 3.390; df = 2; CFI = 0.994; RMSEA = 0.050), and a comparison between our measurement model and a model with unconstrained factor loadings revealed that the latter model did not provide a better fit (Cheung and Rensvold, 2002; Byrne, 2009) with our data [∆χ^2^_(2)_ = 3.390, n.s. and ∆CFI = 0.006]. To evaluate whether aggregating individual task conflict perceptions to the team level was justified, we assessed within-team agreement by calculating mean *r*_wg(j)_ values and ICCs based on a one-way analysis of variance (Bliese, 2000; Klein and Kozlowski, 2000). As indicated above, we excluded teams with mean *r*_wg(j)_ values of lower than 0.50. For the remaining teams, we found a high interrater agreement (mean *r*_wg(j)_ = 0.863; James et al., 1984; LeBreton and Senter, 2008). A one-way analysis of variance and related intraclass correlations (*F* = 3.687, *p* < 0.001; ICC[1] = 0.340; ICC[2] = 0.729) further support the aggregation of team members’ task conflict perceptions.

*High- (*vs. *low-)context culture.* Following prior research (Grandey et al., 2005; Cooper and Watson, 2011; Lai et al., 2022), we used a dummy variable taking a value of 1 for teams operating in the Brazilian high-context culture and 0 for teams operating in the German low-context culture to reflect the cultural differences between the two national context.

*Controls*. In all our analyses, we controlled for *team age*, indicating the average age of team members. Following earlier research (Harrison et al., 1998; Woolley et al., 2010; Apesteguia et al., 2012; Gonzalez-Mulé et al., 2020), we controlled for *team gender*, indicating the percentage of female team members, and *team tenure*, i.e., the average number of years team members had worked on their current team. As larger teams have more potential to break up into subgroups (Shaw, 2004), we also controlled for *team size*. Based on our ASW calculations, we additionally accounted for the *number of subgroups* emerging within teams, which may also affect team outcomes (Carton and Cummings, 2012). Finally, our analyses control for *task interdependence*, i.e., the extent to which team members’ tasks are affected by the work of other team members (Kiggundu, 1981), which may influence team member interactions (Gully et al., 1995). Task interdependence was measured with three items developed by Morgeson and Humphrey (2006). A sample item is “My work tasks are highly dependent on the work of others in my team.” Team members’ answers ranged from 1 (*strongly disagree*) to 5 (*strongly agree*). The items were combined into a single scale (Cronbach’s α = 0.834). Given that we observed a high level of interrater agreement (mean *r*_wg(j)_ = 0.680) and significant between-team variance (*F* = 1.547, *p* = 0.016; ICC[1] = 0.095; ICC[2] = 0.354) for team members’ perceptions of task interdependence, we aggregated them to the team level.

### 3.3. Analytical approach

Overall, our lines of reasoning suggest a model of moderated mediation (Hayes and Rockwood, 2020). We thus conducted a conditional process analysis (Hayes and Rockwood, 2020) and utilized the PROCESS macro—a path analysis modeling tool based on OLS-regression developed by Hayes (2017)—to test our hypotheses. This approach allowed us to not only test for a potential effect of social category faultline strength on task conflict (H1) and whether this effect is contingent upon societal culture (H2), but to also elaborate on whether there is an indirect effect of social category faultline strength on team performance *via* task conflict, which is contingent on societal culture (H3).

Following established recommendations (Hayes, 2017), we calculated coefficients and standard errors for the suggested indirect effects based on a bootstrapping approach. Specifically, we utilized 10,000 bootstrap samples to test the proposed conditional indirect effects and estimate the index of moderated mediation to establish whether there is a significant difference between the indirect effects (Hayes, 2015; Cheung and Lau, 2017).

To facilitate interpretation, we standardized all of our explanatory variables, excluding our binary moderator, before entering them into our analyses.

## 4. Results

Table 1 presents the descriptive statistics and correlations of our study variables.

**Table 1:** Means, standard deviations, and correlations.

Variable	*Mean*	*SD*	1	2	3	4	5	6	7	8	9
1. Team age	39.70	9.14									
2. Team gender	0.64	0.27	−0.313^*^								
3. Team tenure	8.79	6.53	0.688^**^	−0.251^†^							
4. Team size	6.76	3.77	−0.194	0.012	−0.209						
5. Task interdependence	2.47	0.52	−0.451^**^	0.197	−0.305^*^	0.261^†^					
6. Number of subgroups	2.44	0.88	0.099	−0.157	0.002	0.605^**^	0.121				
7. High-context culture^a^	0.41	0.50	−0.806^**^	0.322^*^	−0.524^**^	0.154	0.456^**^	−0.120			
8. Social category faultline strength	0.47	0.26	−0.084	0.146	−0.087	0.553^**^	0.098	0.305^*^	0.023		
9. Task conflict	2.69	0.61	−0.654^**^	0.153	−0.402^**^	0.320^*^	0.518^**^	0.135	0.721^**^	0.183	
10. Team performance	3.93	0.56	−0.201	−0.110	−0.332^*^	0.127	0.053	−0.147	0.000	0.133	−0.096

*N* = 54; *SE* = standard error;

a Dummy coded: 0 = Germany, 1 = Brazil.

† *p* < 0.10;

* *p* < 0.05;

** *p* < 0.01.

Similar to prior research (Valls et al., 2020), Table 1 reveals a significant positive relationship between *social category faultline strength* and *team size* (*r* = 0.553, *p* < 0.001). In addition, we found a significant positive relationship between *social category faultline strength* and the *number of subgroups* (*r* = 0.305, *p* = 0.025). We further observed a significant positive relationship between *high-context culture* and *task conflict* (*r* = 0.721, *p* < 0.001). This is in line with prior research showing that while the societal culture in Brazil emphasizes preserving harmony and discourages open confrontation (Kim et al., 1998), it also creates informality and task ambiguity in the workplace that can stimulate task-related conflict (Bisseling and Sobral, 2011).

Table 2 displays the results of our analyses for testing Hypotheses 1 and 2.

**Table 2:** Results from OLS regression analysis: task conflict.

	Model 1	
	Task Conflict	
Independent variable	Coefficient	SE
Intercept	−0.527^**^	0.152
Team age	−0.143	0.175
Team gender	−0.165^†^	0.097
Team tenure	0.097	0.120
Team size	0.026	0.131
Task interdependence	0.150	0.101
Number of subgroups	0.103	0.115
Social category faultline strength	0.319^*^	0.135
High-context culture^a^	1.308^**^	0.312
Social category faultline strength × high-context culture^a^	−0.497^*^	0.192

*N* = 54; *SE* = standard error;

a Dummy coded: 0 = Germany, 1 = Brazil.

† *p* < 0.10;

* *p* < 0.05;

** *p* < 0.01.

Hypothesis 1 proposes a positive relationship between *social category faultline strength* and *task conflict*. Hypothesis 2 states that societal culture moderates the relationship between *social category faultline strength* and *task conflict* such that this relationship will be weaker in a *high-context culture* than in a *low-context culture*.

**Figure 2:**
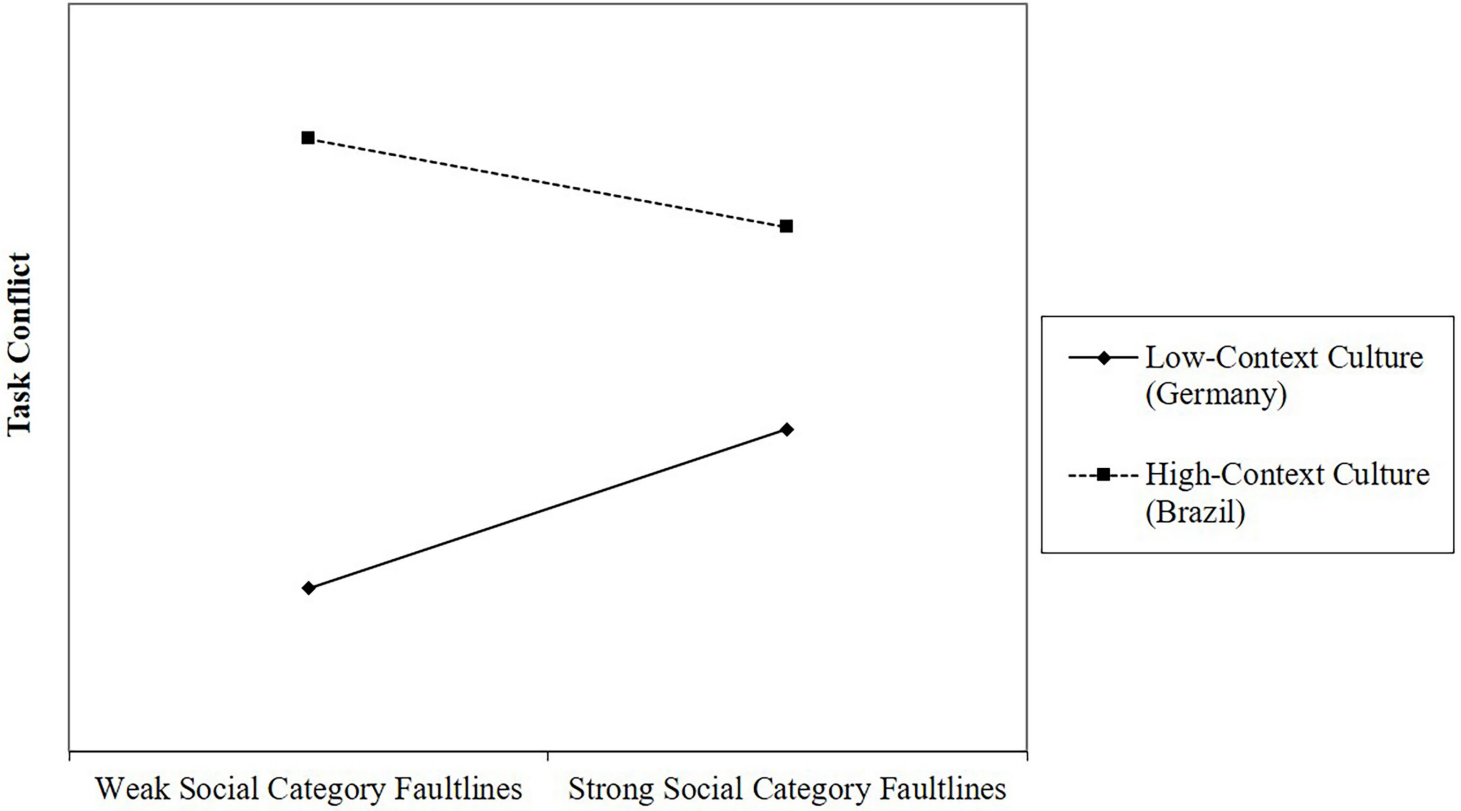
Conditional effects of social category faultline strength on task conflict.

Table 2, Model 1 reveals a positive effect of *social category faultline strength* on *task conflict* (ß = 0.319, *p* = 0.023) and a negative interaction effect of *social category faultline strength* and *high-context culture* (ß = −0.497, *p* = 0.013). To facilitate the interpretation of these results, we probed and plotted the conditional effects of *social category faultlines* on *task conflict* in Germany and Brazil. In support of Hypothesis 3, this analysis revealed a significant positive relationship between *social category faultline strength* and *task conflict* (*b* = 0.319, *p* = 0.023) for teams operating in the *low-context culture* in Germany. In contrast, but in line with Hypothesis 2, we found no significant relationship between *social category faultline strength* and *task conflict* for teams embedded in the *high-context culture* in Brazil (*b* = −0.178, n.s.). Figure 2 shows the conditional effects.

Hypothesis *3* claims that there is a negative indirect relationship between *social category faultline strength* and *team performance* via *task conflict* moderated by societal culture such that this relationship is weaker in a *high-context culture* than in a *low-context culture*.

In line with Hypothesis 3, the results of our analyses shown in Table 3 indicate a significant negative indirect effect of *social category faultline strength* on *team performance via task conflict* for teams operating in the *low-context culture* in Germany (*b* = −0.079, 95% bootstrap CI = −0.190 to −0.014). In contrast, we found no such effect for teams embedded in the *high-context culture* in Brazil (*b* = 0.044, 95% bootstrap CI = −0.029 to 0.158). Lending support for Hypothesis 3, the index of moderated mediation further indicates that the two conditional indirect effects differ significantly (*b* = 0.123, bootstrap 95% CI = 0.022 to 0.299).

**Table 3:** Conditional indirect effects and index of moderated mediation.

	Coefficient	SE (Boot)	95% BootCI	
Low-Context Culture^a^	−0.079	0.046	−0.190	−0.014
High-Context Culture^b^	0.044	0.047	−0.029	0.158
Index of Moderated Mediation^c^	0.123	0.071	0.022	0.299

Number of bootstrap samples = 10,000; SE (Boot) = bootstrap standard error; 95% BootCI = 95% Bootstrap Confidence Interval.

a Germany.

b Brazil.

c Difference between the conditional indirect effects.

## 5. Discussion

We set out to contribute to a better understanding of how the environmental context in which teams operate affects the consequences of social category faultlines. To develop our theoretical reasoning, we integrated ideas from faultline theory (Lau and Murnighan, 1998), Hall’s (1976) theory on cultural contexts, and the input-process-outcome model of team performance (McGrath, 1984).

In line with our theoretical arguments, we find that while social category faultline strength can stimulate task conflict and may thus be detrimental for team performance, these effects heavily depend on the societal culture in which teams are embedded. Specifically, we observe that in the German low-context culture, social category faultline strength stimulates task conflict and thus has a negative indirect effect on how teams perform. In contrast, no such effects can be observed among teams in the Brazilian high-context culture. In the following section, we elaborate on the implications of these findings for faultline research and the literature on task conflicts and high- and low-context cultures before highlighting the practical implications of our results.

### 5.1. Implications for faultline theory and research

Prior scholarly efforts have firmly established that demographic faultlines can be conflictual and thus detrimental for team effectiveness (Thatcher and Patel, 2011; Thatcher and Patel, 2012). However, an emerging stream of research also provides evidence to suggest that to fully understand the consequences of demographic faultlines, one needs to pay attention to the environmental context in which teams operate (Bezrukova et al., 2012; Cooper et al., 2014). In line with this notion, previous studies have shown that cultural alignment between a team and the department in which it is embedded (Bezrukova et al., 2012) as well as characteristics of an organization’s task environment, such as dynamism and complexity (Cooper et al., 2014), can influence the effects emanating from demographic faultlines. Our study reveals that how social category faultlines affect important team processes and outcomes also hinges on whether teams operate in a high- or a low-context culture. With these findings, our study complements previous findings on the interplay between demographic faultlines and teams’ environmental context and contributes to answering related scholarly calls (Gibson and Vermeulen, 2003; Bezrukova et al., 2012; Wu et al., 2021).

Our findings also contribute to an ongoing debate on whether social category faultlines can stimulate task conflict. Meta-analytical evidence (Thatcher and Patel, 2011) suggests that, generally, demographic faultlines stimulate task conflict. In contrast, a study conducted by Choi and Sy (2010) indicates that this inference may need to be refined. Specifically, the authors conclude that while information-based faultlines, i.e., faultlines involving task-related demographic attributes, such as organizational tenure, stimulate task conflict, social category faultlines do not have such an effect. By suggesting that whether social category faultlines play a role in task conflict emergence is contingent upon the cultural context in which teams are embedded, our study further qualifies this conclusion and contributes to a better understanding of the consequences of demographic faultlines.

### 5.2. Implications for task conflict research

Our study may also inform research on the role of team composition in task conflict emergence. Traditionally, scholars have argued that demographic heterogeneity in teams fuels task conflict because it leads to differences in ideas, viewpoints and opinions within a team (Van Knippenberg and Schippers, 2007). However, the results from a meta-analysis conducted by De Wit and Greer (2008) do not fully support this idea, as no significant links between task conflict and team heterogeneity based on demographic attributes such as gender or race could be identified. Jointly with prior research (Bezrukova et al., 2007; Thatcher and Patel, 2011), our study provides evidence in support of the idea that faultlines based on the convergence of social category attributes can be a better predictor of intragroup task conflict than heterogeneity related to a single attribute. However, our study also indicates that whether demographic faultlines result in task conflict or not heavily depends on the societal culture in which teams are embedded. This finding resonates with the idea that differences in societal culture influence social categorization processes and the way members from different subgroups are treated within teams (Schneid et al., 2015), which is crucial for the emergence of task conflict (Molleman, 2005).

Moreover, our study contributes to the ongoing debate on the performance implications of team task conflict (De Dreu and Weingart, 2003; De Wit et al., 2012). While task conflict was originally thought to enhance performance (Jehn, 1995), meta-analytical evidence either suggests task conflict to be negatively related to team performance (De Dreu and Weingart, 2003) or shows no substantial link between the two concepts (De Wit et al., 2012). In line with prior research (Li and Hambrick, 2005; Bezrukova et al., 2007; Jehn and Bezrukova, 2010), we find task conflict emanating from social category faultlines to be detrimental for team performance. This finding indicates that to fully understand the performance implications of task conflict, it may be necessary to consider its causes. By highlighting that the indirect performance effect of social category faultlines conveyed *via* task conflict is affected by differences in societal culture, our study also adds to the debate on whether the link between task conflict and important outcomes is specific to culture (Nibler and Harris, 2003; Bisseling and Sobral, 2011).

### 5.3. Implications for research on high- and low-context cultures

Our study suggests that the differences in the societal norms and values prevalent in high- and low-context cultures serve as crucial contingencies for social categorization processes (Tajfel, 1981; Turner, 2010) and related outcomes. With these findings, our study complements prior research on Hall’s (1976) context theory. Initially, this research in this domain focused on how differences between high- and low-context cultures manifest in how people communicate (Thomas, 1998) and approach and deal with social situations, such as negotiations (Simintiras and Thomas, 1998). More recently, studies have begun to elaborate on whether differences emanating from high- and low-context cultures may affect how individual differences related to personality traits (Mintu-Wimsatt, 2002) and demographic characteristics (Mintu-Wimsatt and Gassenheimer, 2000) influence individual-level outcomes. By highlighting that the differences between high- and low-context cultures also shape the consequences of team composition for team processes and outcomes, our study complements this prior research.

### 5.4. Practical implications

Given that social category faultline strength can be manipulated through selection and placement (Bell, 2007), our study offers important implications for human resource management. Prior faultline research advises managers to ensure a team demographic composition that limits the possibility of homogenous subgroups (Thatcher and Patel, 2011). Our study suggests that this recommendation needs further refinement. Specifically, our findings imply that managers in low-context cultures need to carefully consider the potential negative consequences of social category faultlines and avoid staffing decisions that result in teams being prone to the emergence of homogenous subgroups. If social category faultlines cannot be avoided, these managers may try to alleviate the potential adverse consequences of faultlines by establishing a strong sense of collective identity among team members (Bezrukova et al., 2001). However, our study findings also indicated that managers in high-context cultures may not need to pay particular attention to whether team configurations result in demographic faultlines and may instead focus on other important aspects of team composition.

## 6. Strengths, limitations, and directions for future research

By testing our hypotheses using data from multiple sources, we minimized common-source bias (Podsakoff et al., 2003). Given that our study rests on cross-sectional data, however, we cannot rule out reverse causality, which is a common issue in management research (Aguinis and Edwards, 2014). Thus, we encourage future research to try and replicate our findings based on longitudinal data (Aguinis et al., 2017), which are also well suited to shed more light on how faultline consequences may unfold over time (Schulte et al., 2020). While our sampling approach allows us to compare data from teams operating in very similar industry environments, our study rests on data from just two national contexts. Thus, we cannot rule out that our observations may—at least partially—be attributed to institutional differences that are not subject to our theorizing (Tsui et al., 2007). Thus, we encourage future research to address the interplay between social category faultlines and societal culture based on the data from a much larger number of countries (Gelfand et al., 2017).

Future research may further expand our knowledge of the consequences of demographic faultlines by building on the theoretical arguments and empirical evidence provided in the present study. We focused on the consequences of social category faultlines. Researchers may want to investigate whether differences in societal cultures similarly affect the consequences of information-based faultlines, i.e., faultlines based on task-related demographic attributes, such as education, functional background and company tenure (Bezrukova et al., 2012), as well as faultlines based on deep-level attributes, such as personality traits (Molleman, 2005). Moreover, future research may elaborate on the potential role of faultline activation (Jehn and Bezrukova, 2010) in the interplay between social category faultline strength and societal culture. Specifically, scholars may want to investigate whether the influences emanating from the societal culture in which teams operate are transmitted through emergent states recognized as antecedents of faultline activation, such as team entitlement (Jehn and Bezrukova, 2010).

## Data availability statement

The raw data supporting the conclusions of this article will be made available by the authors, without undue reservation.

## Ethics statement

Ethical review and approval was not required for the study on human participants in accordance with the local legislation and institutional requirements. The patients/participants provided their written informed consent to participate in this study.

## Author contributions

KB and TS contributed to the conception and design of the study. KB collected and analyzed the data and wrote the first draft of the manuscript. TS provided feedback and critical revisions. All authors contributed to the article and approved the submitted version.

## Conflict of interest

The authors declare that the research was conducted in the absence of any commercial or financial relationships that could be construed as a potential conflict of interest.

## Publisher’s note

All claims expressed in this article are solely those of the authors and do not necessarily represent those of their affiliated organizations, or those of the publisher, the editors and the reviewers. Any product that may be evaluated in this article, or claim that may be made by its manufacturer, is not guaranteed or endorsed by the publisher.

## Footnotes

^1^To avoid allocating arbitrary weights to diversity attributes (e.g., a particular difference in age equaling a particular difference in gender distribution), we followed prior research (Bezrukova et al., 2009; Meyer and Glenz, 2018), scaling age by its standard deviation and gender by 1/√2. The latter results in an Euclidean distance of one (Zanutto et al., 2011).
